# Interventions to Promote Patient Utilization of Cardiac Rehabilitation: Cochrane Systematic Review and Meta-Analysis

**DOI:** 10.3390/jcm8020189

**Published:** 2019-02-05

**Authors:** Carolina Santiago de Araújo Pio, Gabriela Chaves, Philippa Davies, Rod Taylor, Sherry Grace

**Affiliations:** 1 School of Kinesiology and Health Science, York University, 4700 Keele St, Toronto, ON M3J 1P3, Canada; carolpio@yorku.ca; 2 Department of Physical Therapy, Federal University of Minas Gerais, Av. Pres. Antônio Carlos, 6627-Pampulha, Belo Horizonte, MG 31270-901, Brazil; gabisschaves@gmail.com; 3 School of Social and Community Medicine, University of Bristol, Queens Road, Bristol BS8 1QU, UK; philippa.davies@bristol.ac.uk; 4 Institute of Health Research, University of Exeter Medical School, St Luke’s Campus, Heavitree Road, Exeter EX1 2LU, UK; rod.taylor@pms.ac.uk; 5 Cardiac Rehabilitation and Secondary Prevention Program, Toronto Rehabilitation Institute, University Health Network, University of Toronto, 399 Bathurst St, Toronto, ON M5T 2S8, Canada

**Keywords:** coronary artery disease, secondary prevention, healthcare access, cardiac rehabilitation

## Abstract

Too few patients utilize cardiac rehabilitation (CR), despite its benefits. The Cochrane review assessing the effectiveness of interventions to increase CR utilization (enrolment, adherence, and completion) was updated. A search was performed through July 2018 of the Cochrane and MEDLINE (Medical Literature Analysis and Retrieval System Online) databases, among other sources. Randomized controlled trials in adults with myocardial infarction, angina, revascularization, or heart failure were included. Interventions had to aim to increase utilization of comprehensive phase II CR. Two authors independently performed all stages of citation processing. Following the random-effects meta-analysis, meta-regression was undertaken to explore the impact of pre-specified factors. Twenty-six trials with 5299 participants were included (35.8% women). Low-quality evidence showed an effect of interventions in increasing enrolment (risk ratio (RR) = 1.27, 95% confidence interval (CI) = 1.13–1.42). Meta-regression analyses suggested that the intervention deliverer (nurse or allied healthcare provider, *p* = 0.02) and delivery format (face-to-face, *p* = 0.01) were influential in increasing enrolment. There was low-quality evidence that interventions to increase adherence were effective (standardized mean difference (SMD) = 0.38, 95% CI = 0.20–0.55), particularly where remotely-offered (SMD = 0.56, 95% CI = 0.36–0.76). There was moderate-quality evidence that interventions to increase program completion were effective (RR = 1.13, 95% CI = 1.02–1.25). There are effective interventions to increase CR utilization, but more research is needed to establish specific, implementable materials and protocols, particularly for completion.

## 1. Introduction

The burden of cardiovascular disease (CVD) is substantial, and it is among the leading causes of disability worldwide [1,2,3]. Cardiac rehabilitation (CR) is a medically-sponsored program to aid recovery and prevent further cardiac events. It includes specific core components that aim to optimize cardiovascular risk reduction, foster healthy behaviors, increase the patient’s understanding of their disease, and improve psychosocial well-being [4,5]. On average, patients attend a program 2 times a week over 5 months [6]. 

CR has been shown to improve quality of life, as well as decrease subsequent morbidity and cardiovascular mortality by approximately 20% [7]. As a result, CR is an integral recommendation in many clinical guidelines for secondary prevention in cardiac patients [8,9,10,11,12,13]. By promoting the utilization of CR, patients can achieve the benefits of participation. Indeed, the more patients participate, the better the outcomes that are achieved [14]. However, CR utilization remains suboptimal. It is estimated that only 30% of eligible patients participate [15,16,17,18]. Such under-utilization can be attributed in part to low referral rates by healthcare providers [19]. However, even among individuals referred to CR, few enroll in the program and many of those who do drop out [20]. Factors impacting utilization of CR include distance, financial resources, work and other time constraints, gender, age, social support, illness perceptions, and depression [21]. 

The 2014 Cochrane systematic review evaluating interventions that promote utilization of CR identified effective interventions to increase CR enrolment [22]. However, this review did not identify sufficient evidence to provide recommendations on interventions to increase adherence; program completion was not considered. Meta-analyses were not undertaken, nor was the quality of evidence rated in accordance with the Grading of Recommendations, Assessment, Development and Evaluation (GRADE) [23]. In recent years, several new trials have been published. The purpose of this study was to undertake an updated systematic review and meta-analysis, applying current Cochrane methodological standards, of interventions to increase patient enrolment, adherence, and completion of CR, as well as to consider equity, costs, and harms.

## 2. Methods

### 2.1. Search Methods for Identification of Studies

The search strategies were designed in accordance with Cochrane Heart Group methods and guidance. A generic search strategy was initially designed, as this review forms part of a broader set of Cochrane CR reviews [7,24,25]. 

The following databases were searched from 2013: (1) Cochrane Central Register of Controlled Trials, July 2018 (Cochrane Library, Wiley, Hoboken, NJ, USA); (2) Database of Abstracts of Reviews of Effects April 2015 (Cochrane Library, Wiley); (3) Health Technology Assessment Database, October 2016 (Cochrane Library, Wiley); (4) MEDLINE through to July 2018 (Ovid, New York, NY, USA); (5) MEDLINE In-Process & Other Non-Indexed Citations through to July 10, 2018 (Ovid) and MEDLINE® Epub Ahead of Print to July 10, 2018 (Ovid); (6) Embase, Embase Classic, and Medline (Elsevier, Amsterdam, Netherlands); (7) Cumulative Index to Nursing and Allied Health Literature (EBSCOhost); and (8) Conference Proceedings Citation Index-Science (CPCI-S) (Web of Science, Clarivate Analytics, Philadelphia, PA, USA).

Reference lists from systematic reviews and meta-analysis (e.g., Matata et al., 2017 [26]) were hand-searched for potentially-relevant articles. Main authors of studies and experts in this field were asked for any missed, unreported, or ongoing trials. Other resources, including trial registers (Clinicaltrials.gov and the World Health Organization International Clinical Trials Registry), were searched to identify recent or ongoing trials. 

### 2.2. Inclusion and Exclusion Criteria

Randomized or quasi-randomized controlled trials (RCTs), either at individual or cluster levels, with either parallel groups or cross-over designs were included. Adults (ages 18 years or over) with myocardial infarction (MI), angina, following coronary artery bypass graft (CABG) surgery, percutaneous coronary intervention (PCI), or with heart failure (HF) who were eligible for CR were included. For studies of enrolment, the study population comprised patients who were eligible for CR. For studies of interventions for increasing adherence or completion, participants were those who had already enrolled to take part in a CR program at the start of the study.

Any interventions with the specific aim of increasing patient utilization of CR were considered. CR programs were defined as those that were comprehensive, phase II (i.e., post-acute care) programs, that offered: (1) Initial patient assessment, (2) prescribed, structured exercise, and (3) at least one other strategy to control CVD risk factors. Interventions could be targeted to individuals, groups, partners, caregivers or other family members, or healthcare professionals. 

Primary outcome measures for this review included: (1) Enrolment in a CR program, defined as patient attendance at a first visit (dichotomous, Y/N), (2) adherence to CR, defined as percentage of total prescribed sessions completed, or (3) completion, where patients attended at least some of the CR intervention components and had a formal re-assessment by the CR team at the conclusion of the program (dichotomous, Y/N) [27]. Trials of adherence or completion had to offer a comparable CR program in the usual care arm. Secondary outcomes included: (1) Harm or adverse events related to the intervention (not CR); (2) costs (i.e., to implement the intervention, or of healthcare avoidance as a result of the intervention), and; (3) equity (i.e., intervention aims to increase utilization in under-represented groups).

### 2.3. Selection of Studies for Inclusion

References identified were imported into Covidence. Two authors (C.P. and G.C.) independently screened titles and abstracts. The full-text reports of potentially-eligible trials were obtained and again these two authors independently assessed them for eligibility. Any disagreements were resolved by discussion or, where agreement could not be reached, by consultation with a third author (S.G.).

### 2.4. Data Extraction and Management

An updated data extraction form based on the one developed for the previous review, the Cochrane Heart group template for RCTs, and the amendments to the protocol for this updated review was developed. Two authors (C.P. and G.C.) independently extracted relevant data characterizing study design, participants, intervention features, risk of bias, and results. One author each transferred extracted data into Review Manager (G.C.) and a statistical software program (C.P.), and second author (C.P. and S.G., respectively) spot-checked the data for accuracy.

### 2.5. Assessment of Potential Bias

At least two authors (C.P. and G.C.) independently assessed the risk of bias in newly-included trials for this update using the Cochrane Collaboration’s recommended tool [28]. Because of the nature of the interventions studied, it was not considered possible to assess the blinding of personnel and participants to treatment assignment (nevertheless this could bias results). Thus, the blinding of outcome assessors was instead considered. Discrepancies were resolved between raters, and ratings were verified by a third author (P.D.). 

We assessed for the presence of publication bias by looking for funnel plot asymmetry and testing this asymmetry using Egger’s test [29]. Finally, one author (CP) used the GRADE Profiler software to assess the overall quality of evidence for each of the 3 outcomes in the review [30]. A second author (PD) checked the assessment.

### 2.6. Data Synthesis and Analysis

Dichotomous outcomes for each comparison have been expressed as risk ratios (RR) with 95% confidence intervals (CI). The continuous outcome of adherence was expressed as standardized mean differences. To perform the meta-analysis, RevMan 5.3 [31] was used. Results were pooled by random-effects meta-analysis with the DerSimonian-Laird method. Heterogeneity amongst included studies was first explored qualitatively by comparing characteristics of included trials and then by visually inspecting forest plots. It was also assessed quantitatively by the Chi2 and I^2^ statistic. I^2^ values around 30–60% were considered indicative of a moderate level of statistical heterogeneity [28], warranting further investigation through random-effects meta-regression.

The univariate meta-regression was undertaken in STATA version 15.1 [32] to explore heterogeneity and examine potential intervention effect modifiers, as pre-specified below. Meta-regression was only performed where at least 10 comparisons were included for an outcome [33]. Given the small number of included trials, it was not considered possible to examine more than one subgroup simultaneously. Given the number of tests performed and hence the potential for error, a more conservative *p*-value of <0.01 was applied (with values < 0.05 considered trends).

The following subgroup analyses were conducted where possible (i.e., sufficient number of trials in each category) to explore significant heterogeneity: (1) Intervention intensity (# of contacts; e.g., mail, visit, calls); (2) intervention deliverer (nurse or allied health care provider vs. other or none); (3) delivery format (any face-to-face vs. no face-to-face); (4) theory-based intervention (yes vs. no); (5) peer navigation (yes vs. no); (6) intervention target (patient vs other); (7) outcome ascertainment (self vs. chart report); (8) multi-center study (multi-site vs. single center); (9) cardiac indication (HF included vs. not included); (10) region (North America vs. other); (11) setting of CR (supervised vs any unsupervised); (12) CR program duration (>3 months vs. <3 months); (13) intervention timing (delivered pre-CR vs. during CR). The last 2 were only considered relevant to the outcomes of adherence and completion. 

## 3. Results

**Figure 1:**
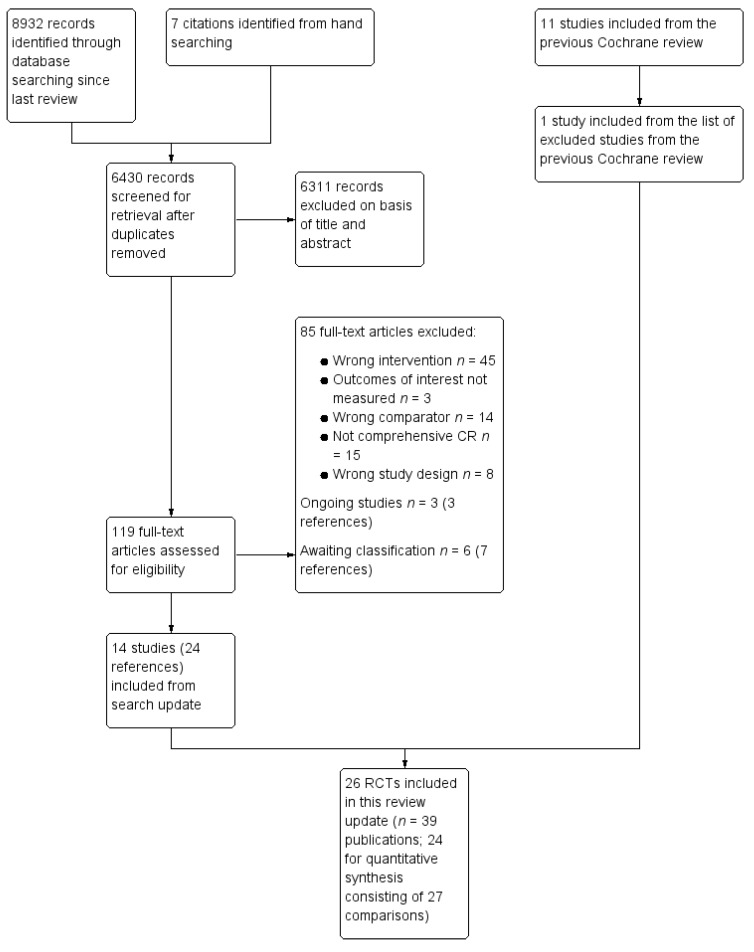
Summary of the study selection process. CR—cardiac rehabilitation; RCTs—randomized or quasi-randomized controlled trials.

The study selection process is illustrated in the flow diagram in Figure 1. The previous version of this Cochrane review [22] included 18 trials, of which 11 were considered eligible for the current review [34,35,36,37,38,39,40,41,42,43,44]. Reasons for exclusion of the 7 trials are shown in Table S1. One previously-excluded study was included in the current review [45]. The updated electronic search yielded 6430 unique citations (Figure 1). Details regarding excluded and ongoing trials, as well as those awaiting classification, are reported elsewhere [46]. Ultimately, 14 new trials met the inclusion criteria [47,48,49,50,51,52,53,54,55,56,57,58,59,60,61]. Thus, 26 trials (5299 participants) have been included in this update; the details of each trial are shown elsewhere [46]. 

### 3.1. Included Studies by Outcome

Sixteen trials (3164 participants) evaluating interventions to increase CR enrolment were included [36,37,38,39,41,42,43,44,45,47,51,55,56,57,58,59]. In all 16, the outcome could be quantified in a manner comparable with the definition used herein, for the purposes of quantitative pooling. Eleven trials (2323 participants) evaluating interventions to increase adherence to CR were included. [34,35,41,48,49,50,51,52,53,54,61,62] Of these RCTs, in 8 (72.7%) the outcome was quantified in a manner comparable with the definition used herein (exceptions were Bertelsen et al. [48], McGrady et al. [61], and Pack et al. [41]). Finally, the outcome of completion was examined for the first time in this review. Seven RCTs (1567 participants) were included for this outcome [34,40,41,49,51,54,59] (8 comparisons). All trials could be included in the quantitative analysis. 

Harms were not measured systematically as a pre-specified outcome for the intervention in any study. Trials may have reported adverse events (or lack thereof) associated with CR participation. Two RCTs included herein incorporated an economic analysis [48,53]. One trial examined the role of home-based CR in increasing adherence, and the other assessed the cost-utility of offering CR in primary care and in the community versus in the hospital.

Six (23.1%) trials applied strategies to increase utilization of CR in previously under-represented patient subsets (i.e., women [35,43,51] and older people [37,45,49]), as per our equity focus. For example, Beckie et al. [35] compared the effect of a gender-tailored CR program with motivational interviewing versus traditional CR on attendance in exercise and education sessions, and Grace et al. [51] compared utilization rates among women referred to supervised mixed-sex (traditional), women-only (not necessarily gender-tailored), or home-based CR. Dolansky et al. [37] studied the effect of a family-directed intervention delivered post-acute care to patients discharged to an inpatient longer-term care facility or receiving home care. Allied healthcare providers in these settings provided cardiac self-management instruction and exercise monitoring.

### 3.2. Characteristics of Included Studies

Trial, patient, CR program, and intervention characteristics in included studies are shown in Table S2. The majority of trials had 2 arms, but one study had 3 arms [51] and one study was a two-by-two factorial design with four arms [55]. One trial was cluster-randomized by general practice [38]. The trial investigator was contacted but could not provide the necessary information to adjust for clustering. This study has contributed to the numerical analysis as if it were individually randomized. The majority (i.e., ≥50%) of participants in twenty-one (80.7%) trials were male, with rates ranging between 66.0% and 87.2% [34,36,38,39,40,41,42,44,47,48,49,50,52,53,54,55,56,57,58,59,61] (Table S2). Most trials included patients with more than one indication for CR (*n* = 22; 84.6%). Please note that Godoy included some primary prevention patients in their sample [50]. 

### 3.3. Intervention Characteristics

The included trials tested a variety of strategies to increase utilization of CR (Table S2 and see Reference [46] for more details). However, the intervention in many trials consisted of contacts by a healthcare provider during or shortly after an acute care hospitalization. For example, several trials utilized a structured telephone call or visit after hospital discharge [36,38,39,43]. Cossette et al. [36] studied the effect of a nursing intervention focused on illness perceptions with a combination of telephone and face-to-face meetings during the 10 days after hospital discharge. Price et al. [43] studied the effects of a nurse-delivered telephone coaching program. McPaul et al. [39] studied the effects of home visits versus telephone follow-up by an occupational therapist on CR attendance. 

In 15 (57.7%) trials, the interventions were theory-based [34,35,36,37,40,43,44,45,49,50,53,54,55,56]. For example, Wyer et al. [44] evaluated the effects of motivational letters based on the Theory of Planned Behavior, and others were based on the Social-Cognitive Theory [43,49,56]. Four (15.4%) trials used peer navigation to promote utilization [42,45,47,57]. In 8 (30.8%) RCTs, the strategy to increase utilization was to offer CR in an unsupervised setting [47,49,50,51,52,53,56,59] (i.e., remotely); in 4 trials, these home-based programs exploited information and communications technology [52,53,56,59]. Overall, the interventions to increase utilization consisted of a mean of 14.5 ± 32.3 contacts. In almost all trials (*n* = 23, 88.5%), the intervention was targeted at the cardiac patient; other targets were nurses, [38] family [37], and groups of patients [49]. In 13 (50.0%) trials, the intervention was delivered pre-CR [36,37,38,39,41,42,43,44,45,47,55,57,58]. 

### 3.4. Risk of Bias in Included Studies

**Figure 2:**
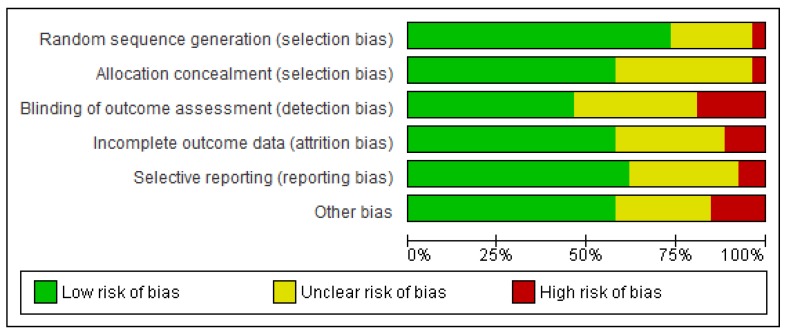
The risk of bias in included trials. Note: Review authors’ judgments about each risk of bias element are presented as percentages across all included trials.

The risk of bias in the 26 included trials given available information is summarized in Figure 2. For 18 (69.2%) trials, the risk was low in 4 or more of the 6 domains. 

Some other potential sources of bias should be considered. First, some trials applied unsupervised programs as a means to increase utilization. These programs do not consist of typical on-site sessions. Therefore, adherence was operationalized as for example completing exercise diaries or logging in to an online system [59]. Thus, in these trials, the operationalization of adherence would be different in both arms (i.e., vs. attendance at supervised sessions). Moreover, it could be argued that completion of online sessions versus going on-site in person for a discharge assessment are not highly comparable. Therefore, results from the trials with unsupervised or hybrid arms [51,53,59] should be considered closely. Second, in the CR4HER trial [51], there was a number of participants that switched treatment groups.

### 3.5. Effects of Interventions

Ultimately 24 (92.30%) trials identified were appropriate for quantitative pooling based on outcome operationalization. A summary of the findings is shown in Table 1. Table 2 shows results of the meta-regression (for enrolment only; there was an insufficient number of comparisons for the other outcomes) where there was a sufficient number of RCTs in each subgroup to run the analysis. 

#### 3.5.1. Enrolment

**Figure 3:**
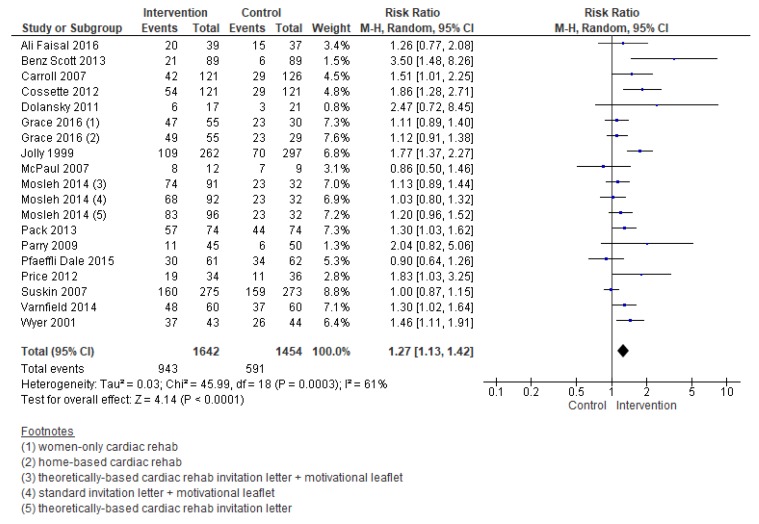
Forest plot summarizing the effect of cardiac rehabilitation utilization interventions on enrollment. Note: Boxes represent the risk ratio (RR) for individual trials. The boxes are proportional to the weight of each study in the analysis and the lines represent their 95% confidence interval (CI). The diamond represents the pooled RR, and its width represents its 95% CI. M-H: Mantel Haenszel method. Tau^2^ represents the variance of the effect size across studies. Chi^2^ (Cochran Q test) represents the weighted sum of squared differences between individual studies and the pooled effect across studies. I^2^ statistic represents the percentage of variation across studies that is due to heterogeneity. Z represents the test for overall effect across all studies. df: degrees of freedom.

Compared with the control, the effect of interventions to increase enrolment were meaningful (RR = 1.27, 95% CI = 1.13–1.42; Figure 3). Heterogeneity was moderate.

**Figure 4:**
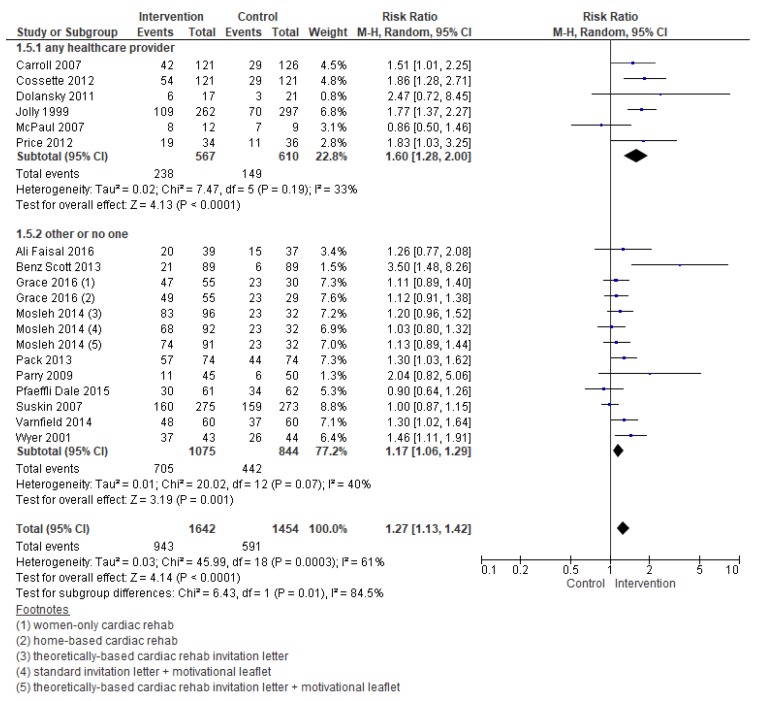
Forest plot for the enrollment—intervention deliverer. Note: Boxes represent the risk ratio (RR) for individual trials. The boxes are proportional to the weight of each study in the analysis and the lines represent their 95% confidence interval (CI). The diamond represents the pooled RR, and its width represents its 95% CI.

**Figure 5:**
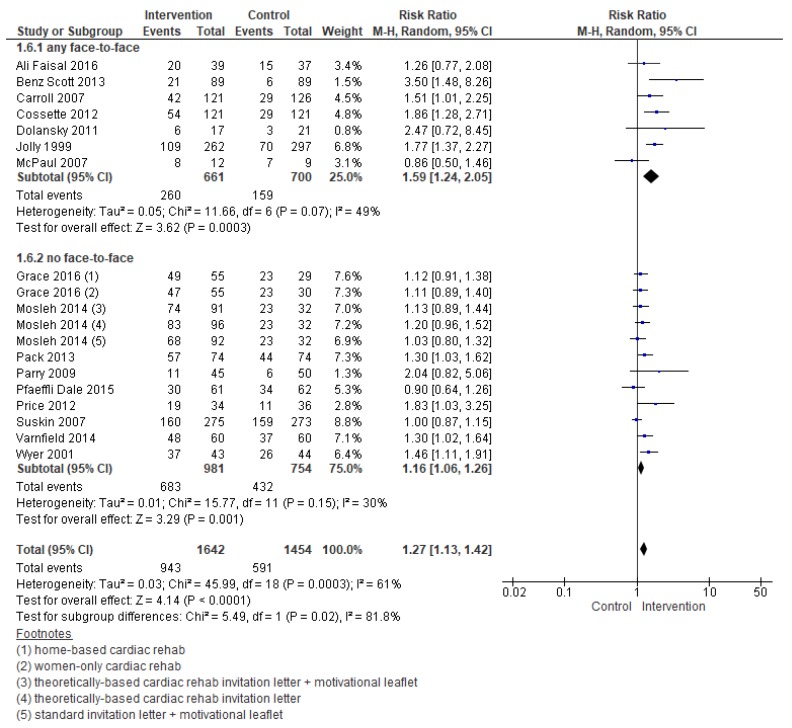
Forest plot for the enrollment—delivery format. Note: Boxes represent the risk ratio (RR) for individual trials. The boxes are proportional to the weight of each study in the analysis and the lines represent their 95% confidence interval (CI). The diamond represents the pooled RR, and its width represents its 95% CI.

Meta-regression analyses revealed the following factors were related to enrolment: Intervention, deliverer, and delivery format (Table 2). Figure 4 and Figure 5 display the forest plots. As shown, interventions targeting healthcare providers and delivered with at least some face-to-face element were more effective. For the other subgroup analyses which could be performed, none were significant.

#### 3.5.2. Adherence

**Figure 6:**
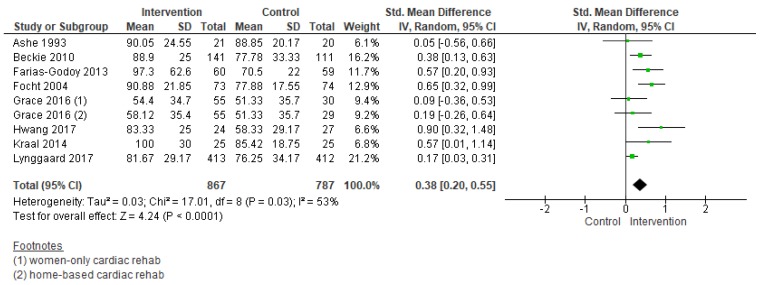
Forest plot summarizing the effect of cardiac rehabilitation utilization interventions on adherence. Note: Boxes represent the standardized mean difference (SMD) for individual trials. The boxes are proportional to the weight of each study in the analysis and the lines represent their 95% confidence interval (CI). The diamond represents the pooled SMD, and its width represents its 95% CI.

**Figure 7:**
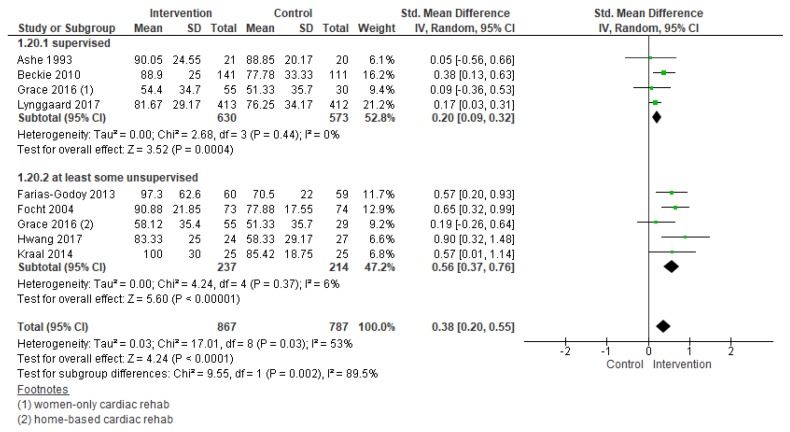
Forest plot for adherence—cardiac rehabilitation setting. Note: Boxes represent the standardized mean difference (SMD) for individual trials. The boxes are proportional to the weight of each study in the analysis and the lines represent their 95% confidence interval (CI). The diamond represents the pooled SMD, and its width represents its 95%.

Results of meta-analysis revealed there was low quality of evidence that interventions to increase adherence had a positive effect (standardized mean difference (SMD) = 0.38, 95% CI = 0.20–0.55; Figure 6). Heterogeneity was moderate. There was an insufficient number of comparisons to undertake meta-regression. Subgroup analyses through meta-analysis revealed interventions in an unsupervised setting (SMD = 0.56, 95% CI = 0.37–0.76; Figure 7) were more effective in increasing adherence. For the other subgroup analyses which could be performed (i.e., intervention deliverer, delivery format, theory-based intervention, multi-center study, cardiac indication, and region), none were significant. 

#### 3.5.3. Completion

**Figure 8:**
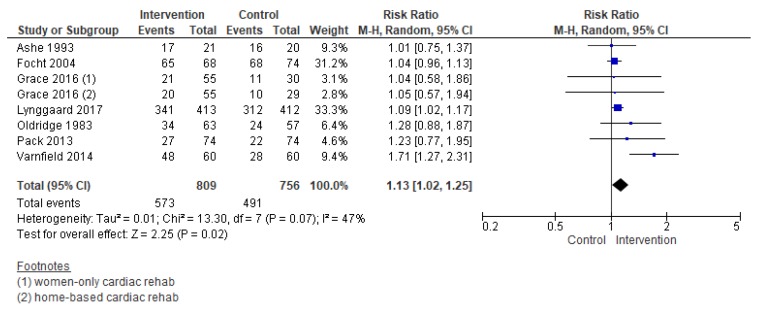
The effect of cardiac rehabilitation utilization interventions on program completion. Note: Boxes represent the risk ratio (RR) for individual trials. The boxes are proportional to the weight of each study in the analysis and the lines represent their 95% confidence interval (CI). The diamond represents the pooled RR, and its width represents its 95% CI.

Compared with control, the effects of interventions to increase CR completion were promising (RR = 1.13, 95% CI = 1.02–1.25, Figure 8). Heterogeneity was moderate. Note in the forest plots that the effect size for Varnfield et al. [59] is considerably larger than the other trials, and could be the source of some of this heterogeneity. Close consideration of the effect of this trial is warranted.

**Figure 9:**
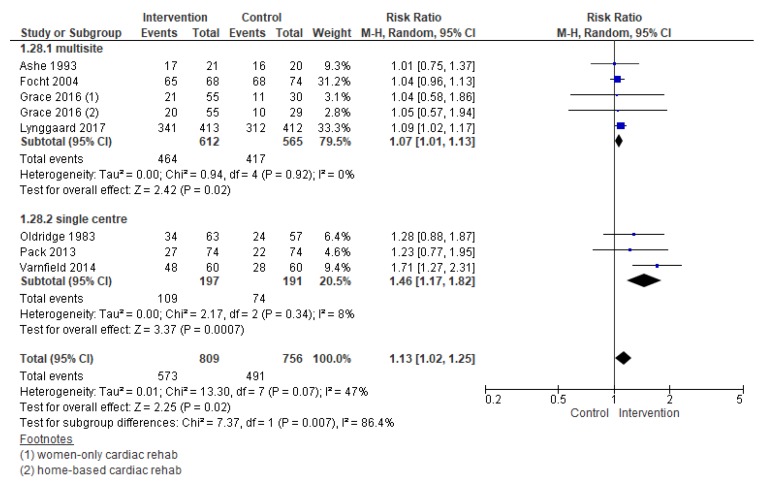
Forest plot for completion—number of sites. Note: Boxes represent the risk ratio (RR) for individual trials. The boxes are proportional to the weight of each study in the analysis and the lines represent their 95% confidence interval (CI). The diamond represents the pooled RR, and its width represents its 95% CI.

Subgroup analysis through meta-analysis (Figure 9) revealed the following factor was related to greater completion: Number of sites. Single-site trials more often resulted in greater completion than multi-site ones, suggesting there may be an issue of generalizability of the interventions tested. For the other subgroup analyses which could be performed (i.e., intervention intensity, intervention deliverer, delivery format, theory-based intervention, intervention target, cardiac indication, region, the setting of CR, intervention timing, and CR program duration), none were significant.

#### 3.5.4. Secondary Outcomes

In both trials reporting on costs, the approach to increase utilization was to deliver CR outside of a hospital setting. In one of the two trials that examined cost [53], it was suggested that home-based CR may be more cost-effective than traditional supervised CR from a societal perspective. (However, the Cochrane review in this area suggests equivalent costs of home versus supervised CR [63]). In the other study [48], average costs to deliver CR in the hospital versus shared between primary care and community were comparable, as were productivity losses in participants in either model. There was suggestion that the shared care model could be cost-effective.

In terms of equity, interventions designed to improve utilization among women [35,43,51] and older patients [37,45,49] were tested, but could not be pooled quantitatively. With regard to the former, results suggest offering alternative models, including women-only programs alone, may not be effective in increasing utilization [51], but tailoring existing models to meet women’s unique needs using a motivational orientation may be [64]. For older participants, peer navigation or post-discharge visits may improve enrolment, and group sessions promoting self-regulation skills may increase completion. No studies compared intervention effects by sub-population.

### 3.6. Publication Bias

**Figure 10:**
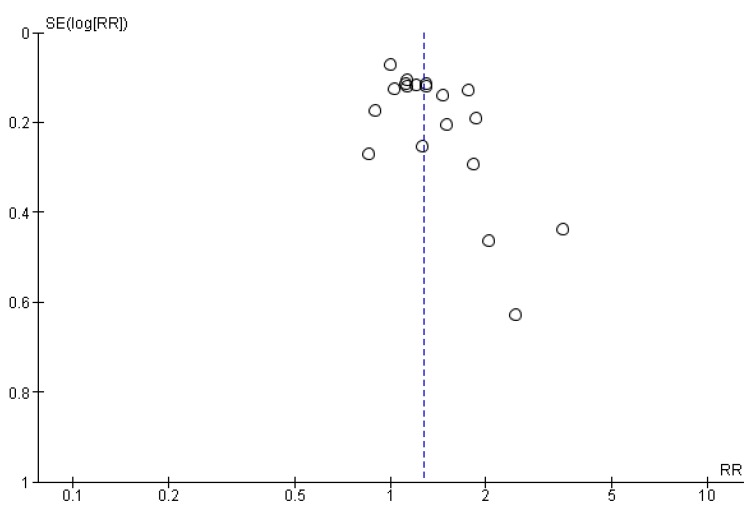
Funnel plot for enrolment. RR - risk ratio; SE - standard error of the estimate.

Funnel plots could not be generated for adherence and completion as there were too few studies. The funnel plot for enrolment is shown in Figure 10. The funnel plot showed a degree of asymmetry, but this was not supported by statistical analysis (Egger’s test, *p* = 0.24). 

### 3.7. Overall Quality of Evidence 

Based on the GRADE method [30], the quality of the evidence was low to moderate for all outcomes (Table 1). The evidence for all outcomes was downgraded due to heterogeneity across studies and indirectness (mostly male samples).

## 4. Discussion

In this first quantitative pooling of randomized trials of interventions to increase CR utilization, it is established that such approaches are indeed successful, resulting in greater enrolment, adherence, and completion than is observed with usual care. There was significant heterogeneity, suggesting some strategies are more effective than others. Enrolment interventions were most successful if delivered by nurses or other allied healthcare professionals (e.g., physiotherapists), face-to-face, whereas for adherence, patients adhered to a greater degree to unsupervised programs. As outlined above, however, adherence ascertainment in supervised and unsupervised settings may not be comparable, therefore these latter findings should be interpreted with caution. Investigating differences in functional capacity in future research may overcome this incomparability. However, CR is shown to be of equivalent efficacy regardless of setting [65], thus if offering CR remotely improved utilization, better outcomes could be achieved. Many programs offer alternative models, but a low proportion of patients are treated in these settings [66]. CR programs must be supported to augment their delivery of alternative models through staffing and reimbursement.

Harms or adverse effects of interventions to increase CR utilization were not considered. An observational study has suggested that offering too much reassurance and optimism to patients about their recovery during CR discussions at the bedside may be associated with less enrolment [67]. While none of the interventions tested in the included studies were associated with significantly lower utilization, clearly the content of structured communications during interventions should be considered, standardized, and tested. 

Healthcare providers need to be aware of the importance of their CR recommendations and provide tangible, simple, and effective strategies to make such recommendations. Indeed, an online course to educate inpatient cardiac care providers on how to discuss CR with patients at the bedside has been developed to promote the implementation of the findings of this review; it is currently being evaluated. Tools and resources from included trials have been collated at [68] http://sgrace.info.yorku.ca/; the Centers for Disease Control Million Hearts Initiative have also collated practical tools [69]. 

Pooling of these diverse interventions is not informative for practice if there is no commonality, understood mechanism, or specific protocol/materials. As there is a good rationale for increasing utilization of CR [7,70], further high-quality research is needed to understand how the interventions work and to ensure they are replicable. The evaluation of single strategies will make it easier to identify the ‘active ingredients’ of interventions. Moreover, there have been other interventions tested in non-randomized studies which warrant testing in RCTs, including systematic referral for augmenting enrolment [51,70], among other quality improvement approaches [41]. 

No trial considered the cost of delivering a utilization intervention specifically. Given the nature of some of the interventions (e.g., healthcare providers making post-discharge home visits), these costs could be considerable and should be quantified in future trials. These costs would substantially impact implementation in the real world. Some tested interventions, however, could be particularly low-cost (e.g., the motivational letter by Wyer 2001 et al. [44]), and hence could be scaled-up across the cardiac population. The costs of intervention delivery should be weighed in relation to the cost-savings associated with CR participation. 

Despite the fact that some included studies considered women and older patients specifically, the majority of participants in the studies included in this review were middle-aged, male, acute coronary syndrome (+/- revascularization) patients. More studies in this review update included HF patients. This is encouraging considering HF is now a recognized indication for CR [24], yet such patients may avoid exercise due to fear of placing excessive strain on the heart or functional limitations. The identification of effective techniques to increase CR utilization in people with HF may, therefore, be particularly valuable.

Intervention effectiveness in under-represented groups such as ethnic minorities, those of low socioeconomic status, and people with comorbidities needs to be tested. Studies did not report intervention effect by these characteristics, and given recommendations for sex and gender-based analyses in particular [71], this should be reported in all future trials. Further trials of gender-tailored CR are needed so that there is sufficient power to test whether they increase utilization or not. Other strategies to increase use in women have been recently reviewed and should perhaps be the subject of an RCT [72]. Despite the many strengths of this review, including the application of GRADE and the fact that this is the first time evidence has been pooled quantitatively, it suffers from some limitations as well. As outlined above, no trials in this area can be double-blinded, and there was a risk of bias in many trials. The evidence may not be applicable to the average cardiac patient. Heterogeneity was also an issue. 

In conclusion, this review shows that a number of different interventions can increase the enrolment in, adherence to, and completion of CR. Interventions to enhance CR enrolment are most effective if delivered face-to-face by healthcare providers. The resource implications of such interventions need careful consideration. Offering unsupervised CR sessions may promote greater adherence. More research is needed to understand specifically how to increase completion and to establish specific, implementable intervention materials and protocols.

## Figures and Tables

**Table 1:** Summary of findings.

Outcomes	№ of Participants (Studies) Follow Up	Certainty of the Evidence (GRADE)	Relative Effect (95% CI)	Anticipated Absolute Effects * (95% CI)	
				Risk with No Interventions to Promote Utilization of CR	Risk Difference WITH Interventions to Promote Utilization of CR
Enrolment	3096 (19 RCTs) – 11 weeks	⊕⊕⊝⊝LOW ^1,2^	RR 1.27 (1.13 to 1.42)	Study population	
				406 per 1000	110 more per 1000 (53 more to 171 more)
Adherence	1654 (9 RCTs) – 18 weeks	⊕⊕⊝⊝LOW ^1,2^	-		SMD 0.38 SD higher (0.20 higher to 0.55 higher)
Completion	1565 (8 RCTs) – 24 weeks	⊕⊕⊕⊝MODERATE ^2^	RR 1.13 (1.02 to 1.25)	Study population	
				649 per 1000	84 more per 1000 (13 more to 162 more)

* The risk in the intervention group (and its 95% confidence interval) is based on the assumed risk in the comparison group and the relative effect of the intervention (and its 95% CI). CI—confidence interval; RCT—randomized controlled trial; RR—risk ratio; SD—standard deviation; SMD—standardized mean difference; CR—cardiac rehabilitation. GRADE Working Group grades of evidence. High certainty (4 ⊕): we are very confident that the true effect lies close to that of the estimate of the effect. Moderate certainty (3 ⊕): we are moderately confident in the effect estimate: the true effect is likely to be close to the estimate of the effect, but there is a possibility that it is substantially different. Low certainty: (1 ⊕) our confidence in the effect estimate is limited: the true effect may be substantially different from the estimate of the effect. Very low certainty (0 ⊕): we have very little confidence in the effect estimate: the true effect is likely to be substantially different from the estimate of effect. ^1^ Heterogeneity suggests evidence of inconsistency, therefore quality of evidence downgraded by one level. ^2^ The included studies consisted of primarily white male participants, therefore quality of evidence downgraded by one level for indirectness.

**Table 2:** Meta-regression results for enrolment.

Subgroup	*n*	Odds Ratio (95% CI)	*p*	Residual I^2^ *
Delivery format (any face-to-face or no face-to-face)	3096	0.73 (0.57 to 0.93)	0.01	37%
Theory-based (yes or no)	3096	0.98 (0.75 to 1.27)	0.86	60%
Outcome ascertainment (self-report or chart-report)	1835	0.99 (0.99 to 1.00)	0.74	53%
Number of sites (multi-site or single-centre)	943	0.90 (0.69 to 1.17)	0.40	60%
Region (North America or other)	3096	0.91 (0.70 to 1.17)	0.44	60%
Intervention Intensity (<5 contacts or ≥5 contacts)	2659	0.99 (0.99 to 1.00)	0.23	66%
Peer navigation (yes or no)	3096	0.74 (0.50 to 1.10)	0.13	55%
Intervention deliverer (nurse or allied health care professional or no one)	3096	0.73 (0.56 to 0.94)	0.02	37%
Intervention target (patient or other)	3096	1.49 (0.98 to 2.28)	0.06	46%
Cardiac indication (heart failure included or not)	2196	0.83 (0.63 to 1.10)	0.19	55%
CR setting (supervised or unsupervised)	1650	1.03 (0.84 to 1.25)	0.76	15%

* I^2^ statistic represents the percentage of variation across studies that is due to heterogeneity. CI—confidence interval; CR—cardiac rehabilitation.

## Supplementary Materials

The following are available online at https://www.mdpi.com/2077-0383/8/2/189/s1, Supplemental Table S1: Excluded studies from previous review, Supplemental Table S2: Summary of characteristics of included trials.
