# Supplementary material for: Interventions to Promote Patient Utilization of Cardiac Rehabilitation: Cochrane Systematic Review and Meta-Analysis
Source: J Clin Med. 2019 Feb 5;8(2):189. doi: 10.3390/jcm8020189 (PMC6406265; doi:10.3390/jcm8020189)
Supplement: Supplementary file 1 [file jcm-08-00189-s001.pdf]

SUPPLEMENTAL MATERIAL

Interventions to promote patient utilization of cardiac rehabilitation: Cochrane systematic review and meta-analysis

p 2	Supplemental Table S1	Excluded studies from previous review
p 3	Supplemental Table S2	Summary of characteristics of included trials

Supplemental Table S1: Excluded studies from previous review

Reference	Exclusion Reason
Arrigo, I.; Brunner-LaRocca, H.; Lefkovits, M.; Pfisterer, M.; Hoffmann, A. Comparative outcome one year after formal cardiac rehabilitation: the effects of a randomized intervention to improve exercise adherence. *Eur. J. Cardiovasc. Prev. Rehabil.* **2008**, *15*, 306–11.	Wrong intervention and outcomes
Daltroy, L. Improving cardiac patient adherence to exercise regimens: a clinical trial of health education. *J. Card. Rehabil.* **1985**, *5*, 40–49.	Not comprehensive CR (i.e. exercise only)
Duncan, K.; Pozehl, B. Effects of an exercise adherence intervention on outcomes in patients with heart failure. *Rehabil. Nurs.* **2003**, *28*, 117–122.	Not comprehensive CR
Hillebrand, T.; Frodermann, H.; Lehr, D.; Wirth, A. Increased participation in coronary groups by means of an outpatient care program. *Herz Kreislauf* **1995**, *27*, 346–349.	Intervention delivered after CR
Izawa, K.P.; Watanabe, S.; Omiya, K.; Hirano, Y.; Oka, K.; Osada, N.; Iijima, S. Effect of the Self-Monitoring Approach on Exercise Maintenance During Cardiac Rehabilitation. *Am. J. Phys. Med. Rehabil.* **2005**, *84*, 313–321.	Intervention delivered after CR
Moore, S.M.; Charvat, J.M.; Gordon, N.H.; Pashkow, F.; Ribisl, P.; Roberts, B.L.; Rocco, M. Effects of a CHANGE intervention to increase exercise maintenance following cardiac events. *Ann. Behav. Med.* **2006**, *31*, 53–62.	Intervention delivered after CR
Sniehotta, F.F.; Scholz, U.; Schwarzer, R. Action plans and coping plans for physical exercise: A longitudinal intervention study in cardiac rehabilitation. *Br. J. Health Psychol.* **2006**, *11*, 23–37.	Not comprehensive CR (i.e. exercise only)

Supplemental Table S2: - Summary of Characteristics of Included Trials

	**n (%) or mean ± standard deviation (median)**
**Study characteristics**	
**Publication year**	2009.9 ± 7.7 (2012.5)
**Study region**	
North America	16 (61.5%)
Europe	3 (11.5%)
Other	7 (27.0%)
**Multi-centre**	14 (53.8%)
**Parallel-group design**	25 (96.1%)
**Sample Size**	193.1 ± 173.1 (135)
**Outcome ascertainment**	
Chart	13 (50.0%)
Self-report	4 (15.4%)
Unclear	9 (34.6%)
**Cardiac Rehabilitation (CR) Program Characteristics**	
**Program Duration (weeks)**	12.8 ± 4.6 (n=10, 38.5% ≥ 3months)
**Women-only**	3 (11.5%)
**Some unsupervised**	8 (30.8%)
**Patient characteristics**	
**Sex**	
Males only	1 (3.9%)
Women only	3 (11.5%)
Mixed-sex	22 (84.6%)
**Age (years)**	63.4 ± 10.4 (median 63.4)

**Diagnosis**<br><br>Any heart failure	 <br>10 (38.4%)
**<u>Intervention Characteristics</u>**	
**Delivered by healthcare provider**	8 (30.7%)
**Some face-to-face**	14 (53.8%)
**Number of contacts**	12.7 ± 32.0
**Delivered Pre-CR**	13 (50.0%)
**Target – Patient**	23 (88.5%)
